# Endoplasmic reticulum stress in diethylnitrosamine-induced rat liver cancer

**DOI:** 10.3892/ol.2013.1651

**Published:** 2013-10-29

**Authors:** BIN XIAO, LI-MIN CUI, DONG-JIE MA, SHUANG-PING LIU, XUE-WU ZHANG

**Affiliations:** 1 College of Medicine, Yanbian University, Yanji, Jilin 133002, P.R. China; 2 Affiliated Hospital of Yanbian University, Yanji, Jilin 133002, P.R. China

**Keywords:** endoplasmic reticulum stress, diethylnitrosamine, rats, liver cancer

## Abstract

To analyze the significance of endoplasmic reticulum stress (ERS) in the development of diethylnitrosamine (DEN)-induced liver cancer in rats, critical regulatory factors in ERS signaling pathways were investigated in the present study. The results showed that the expression of ERS-related proteins gradually increased in the early and mid-term stages of carcinogenesis, while in the later stages, the expression of these proteins did not change significantly after reaching a peak. ERS is involved in DEN-induced rat liver injury, the proliferation of liver cells and the occurrence and development of liver cirrhosis. However, ERS did not affect hepatoma cell growth following the formation of rat liver cancer in the current study.

## Introduction

The endoplasmic reticulum (ER) is an organelle that regulates the synthesis, folding and aggregation of intracellular proteins. ER stress (ERS) is a subcellular organelle pathology in which the aggregation of unfolded or misfolded proteins in the ER leads to its dysfunction. Previous studies have highlighted evidence that ERS is closely associated with viral hepatitis, non-alcoholic fatty liver disease, alcoholic liver damage, liver cancer and other liver diseases (1–6).

The unfolded protein response (UPR) is a protective response mediated by the ER chaperone, glucose-regulated protein 78 (GRP78), and three ERS receptor proteins, protein kinase-like ER kinase (PERK), activating transcription factor 6 (ATF6) and inositol-requiring enzyme 1 (IRE1). If ERS is absent, PERK, ATF6 and IRE1 combine with GRP78, making it inactive. When ERS is present, GRP78 dissociates from these three transmembrane proteins in favor of combining with the unfolded protein. Following dissociation, the receptor proteins are activated and initiate the UPR, which may raise the expression of GRP78 and folded protease by inhibiting protein synthesis. In addition, the receptor proteins promote ER-associated degradation to reduce the aggregation of unfolded or misfolded proteins in the ER, protect the cell from ERS-induced damage and restore normal cell function. In addition to initiating the ERS-mediated adaptive response when ERS is marked or long-term, PERK, ATF6 and IRE1 initiate ERS-mediated apoptosis, inducing cell damage and apoptosis. Previous studies have indicated that ERS induces apoptosis through the following pathways: CCAAT/enhancer-binding protein homologous protein (CHOP); growth arrest/DNA damage-inducible protein 153; C-Jun N-terminal kinase (JNK); and caspase (7).

In the current study, intermittently administrated diethylnitrosamine (DEN) was used to induce a rat liver cancer model that simulated the occurrence and development of human liver cancer. The critical regulatory factors in three ERS signaling pathways were observed during the progression of hepatocellular carcinoma (HCC) in order to clarify the mechanism of liver cancer and to provide an experimental basis for its prevention and targeted therapy.

## Materials and methods

### Rat liver cancer model

In total, 136 male, 5-week-old Wistar rats [SCXK-(Ji) 2007-0003; Experimental Animal Center of Bethune Medical College of Jilin University, Certificate of Conformity, Yanji, China] with body weights of 140–160 g and which had been feeding stably for 7 days, were divided into experimental and control groups. The experimental group (n=120) was provided with sterile drinking water containing 0.01% DEN (purity, 99.9%; Sigma-Aldrich, St. Louis, MO, USA) *ad libitum*. The water containing DEN was replaced every day. After 5 weeks, the rats were provided with DEN-free water for three weeks and then 0.01% DEN solution for 12 weeks prior to withdrawal of the drug. The control group (n=16) received sterilized drinking water without DEN for the duration of the experiment. In total, 15 experimental rats were sacrificed at 5, 8, 10, 12, 14, 16, 18 and 20 weeks each, respectively, with two control rats of the same age sacrificed at each of these time-points. This study was approved by the ethics committee of Yanbian University (Yanji, China).

### Specimen collection and processing

Experimental rats were sacrificed, and the appearance, color and texture of the livers were recorded. Specific sections of liver or liver cancer tissues were fixed in 4% paraformaldehyde, paraffin-embedded and sectioned for HE staining. Other sections of the liver or liver cancer tissues (1×1×1 mm) were fixed in 2.5% glutaraldehyde at 4°C, rinsed twice in 0.1 mol/l PBS and then fixed in 1.0% osmium tetroxide and embedded in EPON812 for ultra-thin sections, which were double-stained with uranyl acetate and lead citrate and observed by a JEM1200EX transmission electron microscope (JEOL, Tokyo, Japan).

### Western blotting

Livers and tumors were lysed in lysis buffer (Pierce Biotechnology, Inc., Rockford, IL, USA) and then centrifuged at 12,000 × g for 15 min. Protein concentration was determined using the BCA kit (Pierce Biotechnology, Inc.) according to the manufacturer’s instructions. A 70-μg protein sample was fractionated by 10% sodium dodecyl sulfate polyacrylamide gel electrophoresis and transferred to a polyvinylidene fluoride membrane (Pall Corporation, Port Washington, NY, USA). Following blocking for 1 h with 5% milk in Tris-buffered saline and Tween-20, the following primary antibodies were added and the blots were incubated at 4°C overnight: Rabbit polyclonal anti-GRP78, rabbit polyclonal anti-PERK, rabbit polyclonal anti-ATF6, rabbit monoclonal anti-IRE-1, goat monoclonal anti-CHOP, rabbit polyclonal anti-eIF2α and rabbit monoclonal anti-TRAF2 or anti-caspase-12 (1:400; Boshide Biotechnology Co., Ltd., Wuhan, China). Following incubation with secondary antibodies (1:5,000), the membranes were visualized by chemiluminescence. The intensity of the protein bands was quantitatively determined using an ultraviolet crosslinker (Bio-Rad, Hercules, CA, USA) and normalized with the intensity of the actin (rabbit polyclonal anti-calnexin; Nanjing KeyGen Biotech., Co., Ltd., Nanjing, China) band in each gel.

### Quantitative (q)PCR

Total RNA was extracted from the tumors using the RNeasy Plus Mini kit (Nanjing KeyGen Biotech., Co., Ltd.) according to the manufacturer’s instructions. cDNA was generated with the iScript Select cDNA Synthesis kit and analyzed by qPCR using SyberGreen qPCR primer assays and the iCycler iQ multicolor real-time PCR detection system (all Nanjing KeyGen Biotech., Co., Ltd.). Relative expression levels were normalized against β-actin expression run concurrently as a reference control. Primer sequences are shown in Table I.

### Statistical analysis

Data from all experiments are presented as the mean ± SD. Statistical differences were evaluated using one-way ANOVA and independent t-tests of sample pairs with SPSS 13.0 software (SPSS, Inc., Chicago, IL, USA). P<0.05 was considered to indicate a statistically significant difference.

## Results

### Rat liver pathological changes

#### Control rats

**Figure 1:**
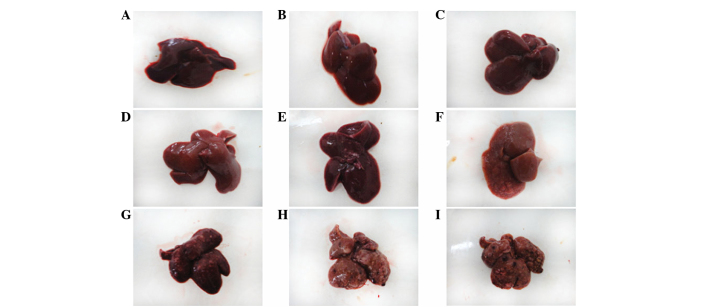
General changes in liver tissue during the development of DEN-induced rat liver cancer. (A) Normal group, the liver exhibited a smooth, glossy surface and a soft texture. Experimental group following the induction of rat liver cancer at (B) 5 weeks; (C) 8 weeks, the liver was of normal appearance; (D) 10 weeks; (E) 12 weeks; (F) 14 weeks, the liver exhibited a rough surface with gray lesions; (G) 16 weeks; (H) 18 weeks; and (I) 20 weeks, the surface of the liver was covered with nodules of various sizes. DEN, diethylnitrosamine.

**Figure 2:**
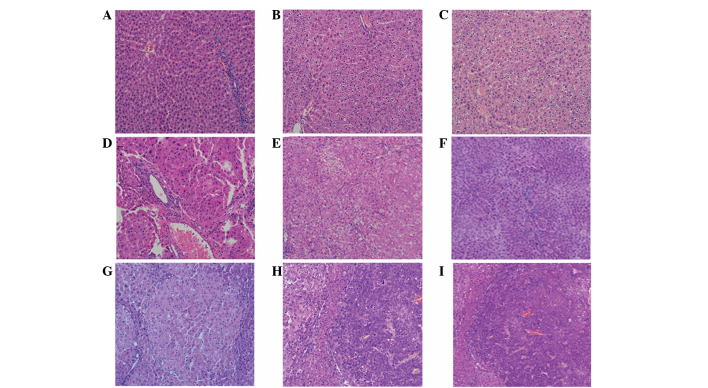
Changes in liver tissue during the development of DEN-induced rat liver cancer, as observed by light microscopy. (A) Normal group, the architecture of the hepatic lobes was complete, with hepatocytes arranged in neat rows and clear cell nuclei. Experimental group following the induction of rat liver cancer at (B) 5 weeks; (C) 8 weeks, the architecture of the hepatic lobes was basically complete and there was visible intralobular focal necrosis with infiltrating inflammatory cells; (D) 10 weeks; (E) 12 weeks; (F) 14 weeks, the architecture of the hepatic lobes was damaged and the hepatocytes were proliferating, which was accompanied by severe steatosis and the formation of visible pseudolobules; (G) 16 weeks; (H) 18 weeks; and (I) 20 weeks, the cancer cells exhibited evident atypia, with abnormally large nuclei and diminished cytoplasm (HE staining; magnification, ×200). DEN, diethylnitrosamine.

**Figure 3:**
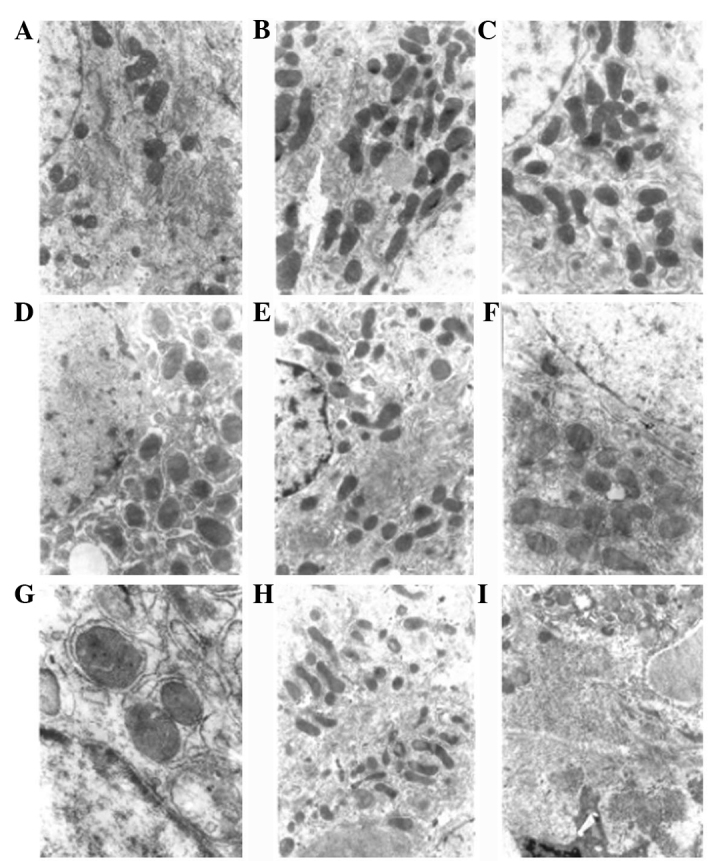
Electron microscopy showing changes in liver tissue in DEN-induced rat liver cancer. (A) Normal group, the hepatocytes were round or oval and in regular arrays, the ratio of nucleus to cytoplasm was normal and cytoplasmic organelles were abundant. Experimental group following the induction of rat liver cancer at (B) 5 weeks; (C) 8 weeks, the hepatocytes were swollen, with swollen mitochondria, the granular matrix had disappeared and there was granulovacuolar degeneration; (D) 10 weeks; (E) 12 weeks; (F) 14 weeks, aggregation of hepatocyte chromatin, increased number of mitochondria, distrupted cristae of the mitochondria, dilation of the rough ER, uneven nuclear membranes and migration of the nucleoli to the side of the cell was observed; (G) 16 weeks; (H) 18 weeks; and (I) 20 weeks, larger hepatocyte nuclei, reduced number of mitochondria, disrupted structure of the mitochondria, loss of the layered structure of the rough ER, fragmented plasma membrane and dissociation of the ribosomes from the rough ER was observed (TEM; magnification, ×6,000). DEN, diethylnitrosamine; ER, endoplasmic reticulum.

The surface of the liver was smooth and brown with an evident gloss, and the texture was soft (Fig. 1A). Observed by light microscopy, the structure of the hepatic lobule was complete, with hepatocytes arranged in neat rows and clear nuclei (Fig. 2A). By electron microscopy, the cell morphology was regular, rounded or oval and the ratio of nucleus to cytoplasm was normal. Cytoplasmic organelles were abundant, with well-developed mitochondria, rough and smooth ER and Golgi complexes (Fig. 3A).

#### Experimental rats

The experimental rat liver pathology was divided into the following three temporal stages: Early carcinogenesis-hepatocyte injury period (1–8 weeks); interim carcinogenesis-sclerosis (9–14 weeks); and late carcinogenesis-cancer period (15–20 weeks). In the early carcinogenesis-hepatocyte injury period, the appearance of the liver was not evidently abnormal (Fig. 1B and C). Observed by light microscopy, the architecture of the hepatic lobes was basically complete, although a few of the cells exhibited ballooning degeneration. There was visible intralobular focal necrosis with inflammatory cell infiltration and gradual emergence of fibrous tissue proliferation and regeneration of hepatocytes (Fig. 2B and C). Electron microscopy observed swelling hepatocytes, with swelling mitochondria and the disappearance of the granular matrix, which was accompanied by granulovacuolar degeneration (Fig. 3B and C). In the interim carcinogenesis-sclerosis period (9–14 weeks), the surface of the liver gradually roughened and varying numbers of large and small gray lesions appeared (Fig. 1D–F). As observed by light microscopy, the normal lobular structure was destroyed, hepatocytes were replaced by fibrous tissue and the typical pseudolobular structure had formed (Fig. 2D–F). Electron microscopy revealed the aggregation of hepatocyte chromatin, increased numbers of mitochondria, disrupted mitochondrial cristae, dilated rough ER, uneven nuclear membranes and the migration of nucleoli to the edges of the cells (Fig. 3D–F). In the late carcinogenesis-cancer period (15–20 weeks), the surface of the liver was covered with multiple large and small nodules (Fig. 1H–J). Observed by light microscopy, the cancer cells exhibited evident atypia, with larger nuclei and less cytoplasm than normal, and there were a number of monocytes and mitotic figures (Fig. 2H–J). Electron microscopy showed hepatocyte nuclei of an increased size, fewer mitochondria, a disrupted mitochondrial structure, loss of the layered structure of the rough ER, fragmentation of the plasma membrane and dissociation of the ribosomes from the ER (Fig. 3H–J).

### Test results of associated factors in the ERS reaction pathway

**Figure 4:**
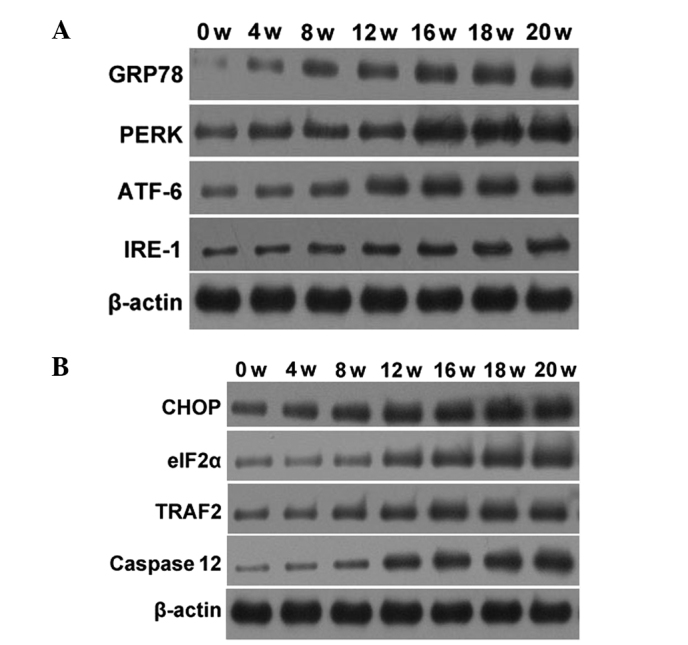
Western blot analysis of (A) UPR-related proteins expressed during ERS and (B) proteins associated with three apoptotic pathways induced by ERS in DEN-induced rat liver cancer tissue. UPR, unfolded-protein response; ERS, endoplasmic reticulum stress; DEN, diethylnitrosamine; GRP78, glucose-regulated protein 78; PERK, protein kinase-like endoplasmic reticulum kinase; ATF-6, activating transcription factor 6; IRE-1, inositol-requiring enzyme 1; CHOP, CCAAT/enhancer-binding protein homologous protein.

**Figure 5:**
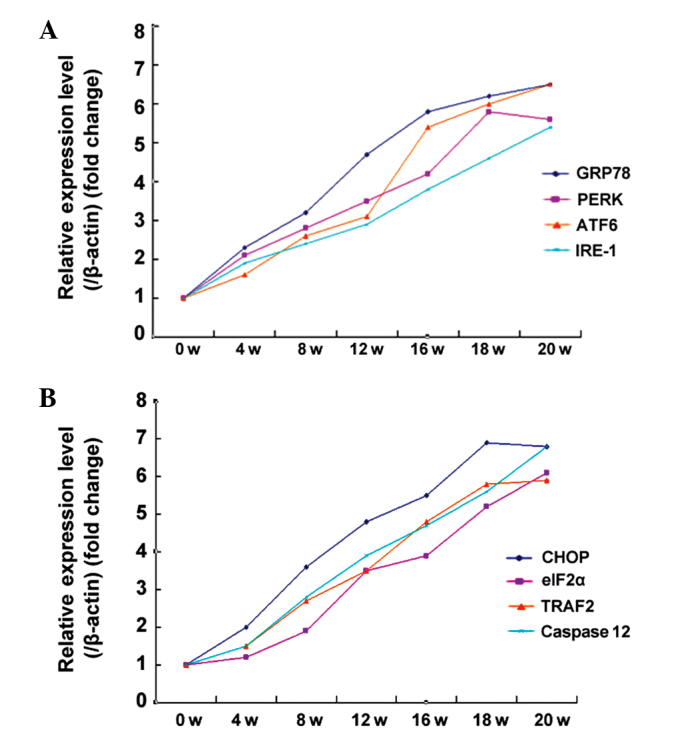
qPCR of (A) UPR-related genes expressed during ERS and (B) genes associated with three apoptotic pathways induced by ERS in DEN-induced rat liver cancer tissue. qPCR, quantitative PCR; UPR, unfolded-protein response; ERS, endoplasmic reticulum stress; DEN, diethylnitrosamine; GRP78, glucose-regulated protein 78; PERK, protein kinase-like endoplasmic reticulum kinase; ATF-6, activating transcription factor 6; IRE-1, inositol-requiring enzyme 1; CHOP, CCAAT/enhancer-binding protein homologous protein.

The expression of the GRP78, PERK, ATF6 and IRE-1 proteins was tested in the UPR associated with the ERS reaction pathway. During early and mid-term carcinogenesis (1–14 weeks), the expression of these proteins gradually increased. In late carcinogenesis (15–20 weeks), the protein expression was essentially constant subsequent to reaching a peak. The western blotting results are shown in Fig. 4A. The expression of CHOP, eIF2α, TRAF2 or caspase-12 proteins, involved in the three pathways that induce apoptosis by ERS, were further tested. During early and mid-term carcinogenesis (1–14 weeks), the expression of these proteins gradually increased, and in late carcinogenesis (15–20 weeks) the expression of the proteins was essentially constant subsequent to reaching a peak. The western blotting results are shown in Fig. 4B. In order to explore any changes in the transcriptional levels of ERS-associated factors, qPCR was used to assess their RNA levels. The observed changes in the RNA levels of GRP78, PERK, ATF6, IRE-1, CHOP, eIF2α and TRAF2 or caspase-12 were consistent with the protein expression results (Fig. 5).

## Discussion

Human hepatoma evolves in the following multi-stage process: i) Damage due to hepatitis B or C viral infection causing chronic hepatitis or cirrhosis; ii) adenomatous hyperplasia nodules; iii) early HCC; iv) advanced HCC; and v) HCC metastasis. This multi-stage occurrence and development model has been confirmed by pathological and clinical cases. In the present study, the modified intermittent administration method, developed by Zhang *et al*(7), was used to successfully induce the hepatoma model in Wistar rats. The model is simple, with a short tumorigenic cycle, and the pathological process follows the general development of human liver cancer. The results of the current study are similar to those described previously (7), indicating that the model is stable and has good reproducibility.

ER dysfunction is likely to lead to the accumulation of misfolded and unfolded proteins in the ER lumen, thereby causing ERS. Short-term ERS protects cells, but when ERS persists for a prolonged duration or is highly intensive, unfolded proteins accumulate in the lumen of the ER, leading to the dissociation of the GRP78/BIP chaperone and the transmembrane proteins, PERK, ATF6 and IRE-1, thus inducing apoptosis (8,9). ERS induces apoptosis through the activation of the following three pathways (10,11): i) CHOP, when ERS persists for a prolonged duration the translation initiation factor, eIF2α, is phosphorylated, which directly stimulates the translation of associated proteins, causing the activation of transmembrane proteins, ATF6 and IRE-1, which induce the transcription and expression of CHOP, leading to apoptosis; ii) ASK1/JNK, ERS activates IRE-1, which binds with TRAF2 to activate ASK1 and further activate JNK, which induces apoptosis; and iii) caspase, when ERS occurs, caspase-12 is activated through a variety of mechanisms to initiate the downstream caspase-3-mediated apoptosis pathway.

In the present study, the ERS components and downstream regulatory factors were assayed at the protein and RNA levels. It was found that during early and medium-term carcinogenesis (1–14 weeks), the expression of GRP78, PERK, ATF6 and IRE-1, involved in the UPR, as well as CHOP, eIF2α and TRAF2 or caspase-12, associated with apoptosis, gradually increased. These results indicated that during DEN-induced rat liver injury, liver cell proliferation and cirrhosis, the liver undergoes the ERS reaction, and ERS is involved in each of these processes. No significant changes in the expression of ERS-associated proteins were identified in the late stage of carcinogenesis (15–20 weeks) once it reached a peak. This indicated that ERS may not be involved in the growth of liver cancer cells following the initial formation of DEN-induced rat liver cancer. This hypothesis remains to be confirmed. The present study shows that ERS is involved in the occurrence and development of primary liver cancer, highlighting a new line of thinking for future studies and the treatment of liver diseases.

## Figures and Tables

**Table I:** Primer sequences.

Target gene	Sequence 5′-3′	Length, bp
β-actin	F: GCAGAAGGAGATTACTGCCCT R: GCTGATCCACATCTGCTGGAA	136
GRP78	F: TCGACTTGGGGACCACCTAT R: GCCCTGATCGTTGGCTATGA	77
PERK	F: GAAGTGGCAAGAGGAGATGG R: GAGTGGCCAGTCTGTGCTTT	61
ATF6	F: GGACCAGGTGGTGTCAGAG R: GACAGCTCTGCGCTTTGGG	61
IRE-1	F: TCATCTGGCCTCTTCTCTCGGA R: TTGAGTGAGTGGTTGGAGGC	77
CHOP	F: ACCACCACACCTGAAAGCAG R: AGCTGGACACTGTCTCAAAG	86
eIF2α	F: TTGAACTGTTGTGACCCCGAC R: CGTAGTCTGCCCGATTTTGC	71
TRAF2	F: TGCTATCTTCTCCCCAGCCT R: TCGCCATTCAAGTAGACCCG	75
Caspase-12	F: TGGATACTCAGTGGTGATAA R: ACGGCCAGCAAACTTCATTA	76

F, forward; R, reverse; GRP78, glucose-regulated protein 78; PERK, protein kinase-like endoplasmic reticulum kinase; ATF-6, activating transcription factor 6; IRE-1, inositol-requiring enzyme 1; CHOP, CCAAT/enhancer-binding protein homologous protein; eIF2α, eukaryotic translation initiation factor 2A; TRAF2, TNF receptor-associated factor 2; caspase-12, cysteinyl aspartate specific proteinase 12.
